# The use of P300-based BCIs in amyotrophic lateral sclerosis: from augmentative and alternative communication to cognitive assessment

**DOI:** 10.1002/brb3.57

**Published:** 2012-07

**Authors:** Pietro Cipresso, Laura Carelli, Federica Solca, Daniela Meazzi, Paolo Meriggi, Barbara Poletti, Dorothée Lulé, Albert C Ludolph, Vincenzo Silani, Giuseppe Riva

**Affiliations:** 1 Applied Technology for Neuro-Psychology Lab, IRCCS Istituto Auxologico ItalianoMilan, Italy; 2 Department of Neurology and Laboratory of Neuroscience - “Dino Ferrari” Center - Università degli Studi di Milano - IRCCS Istituto Auxologico ItalianoMilano, Italy; 3 Polo Tecnologico–Biomedical Technology Department, Fondazione Don Carlo Gnocchi OnlusMilano, Italy; 4 Department of Neurology - University of UlmUlm, Germany

**Keywords:** Amyotrophic lateral sclerosis, augmentative and alternative communication, brain-computer interface, cognitive assessment, P300

## Abstract

The use of augmentative and alternative communication (AAC) tools in patients with amyotrophic lateral sclerosis (ALS), as effective means to compensate for the progressive loss of verbal and gestural communication, has been deeply investigated in the recent literature. The development of advanced AAC systems, such as eye-tracking (ET) and brain-computer interface (BCI) devices, allowed to bypass the important motor difficulties present in ALS patients. In particular, BCIs could be used in moderate to severe stages of the disease, since they do not require preserved ocular-motor ability, which is necessary for ET applications. Furthermore, some studies have proved the reliability of BCIs, regardless of the severity of the disease and the level of physical decline. However, the use of BCI in ALS patients still shows some limitations, related to both technical and neuropsychological issues. In particular, a range of cognitive deficits in most ALS patients have been observed. At the moment, no effective verbal-motor free measures are available for the evaluation of ALS patients’ cognitive integrity; BCIs could offer a new possibility to administer cognitive tasks without the need of verbal or motor responses, as highlighted by preliminary studies in this field. In this review, we outline the essential features of BCIs systems, considering advantages and challenges of these tools with regard to ALS patients and the main applications developed in this field. We then outline the main findings with regard to cognitive deficits observed in ALS and some preliminary attempts to evaluate them by means of BCIs. The definition of specific cognitive profiles could help to draw flexible approaches tailored on patients’ needs. It could improve BCIs efficacy and reduce patients’ efforts. Finally, we handle the open question, represented by the use of BCIs with totally locked in patients, who seem unable to reliably learn to use such tool.

## Introduction

Amyotrophic lateral sclerosis (ALS) is a progressive neurodegenerative disease involving motor neurons in the cerebral cortex, corticospinal tract, brainstem, and spinal cord. Clinically, patients show signs and symptoms of upper and lower motor neuron disease, with spasticity and hyperreflexia corresponding to the former, and fasciculations, weakness and muscle wasting corresponding to the latter condition. Two different types of onset are mainly distinguishable: the spinal onset, with patients presenting initially with weakness and atrophy distally in one limb and the bulbar one, characterized by severe dysarthria and dysphagia. In addition to motor symptoms, cognitive impairment, especially involving frontal executive functions, is a typical feature of the disease. Also “pseudobulbar” symptoms such as emotional lability, with difficulties in controlling episodes of laughing or crying, are seen in a significant number of cases (Gallagher 1989). Usually, respiratory difficulties develop insidiously during the course of the disease, causing dyspnoea and orthopnea, which require either noninvasive or invasive ventilation. As disease progresses, the weakness becomes more widespread, mobility and function of upper limbs undergo a decline and patients may become quadriplegic. Patients gradually lose the ability to articulate words and phrases up to the total loss of verbal communication, that is, anarthria. Moreover, since also limbs mobility is impaired, it deprives patients of the ability to use gestural communication.

Since patients may completely lose the ability to communicate, they could gain enormous benefit from technical support and augmentative communication strategies to continue communication, despite the physical impairment that otherwise would prevent it.

Thus, several questions arise: is communication possible despite motor and cognitive impairments in ALS patients? Moreover, is technology at hand to ensure patients a good quality of communication? In this work, we attempt to answer these questions with a literature-based approach, trying furthermore to make some consideration about future challenges.

Technology represents nowadays a common approach to give and/or improve communication in ALS patients. In particular, augmentative and alternative communication (AAC) can be considered as a form of compensation, which aims to help and improve communication abilities of individuals with difficulties in using common channels of communication, especially verbal and written. The AAC systems are defined augmentative because they extend or may even replace means of communication for physically impaired people. At the same time, they are defined alternative as they use multimodal methods of communication, which are different from the traditional ones. AAC aims to compensate for a temporary or permanent disability of communication (both verbal and written or gestural) and it gives patients the opportunity to maintain their communicative function by producing written or spoken messages, adopting methodologies that range from simple technology (as alphabetic tables) to high-tech computer systems, such as eye-tracking (ET) and brain-computer interface (BCI) devices. The development of these advanced AAC systems allow to bypass the motor difficulties present in ALS patients. In particular, BCIs could be used also in moderate to severe stages of the disease, since they do not require preserved ocular-motor ability, which is necessary for ET applications.

BCI uses neurophysiological signals as input commands to control external devices, bypassing motor output, and conveying messages directly from the brain to a computer. Despite the advantages provided by BCI systems, to date they show some limitations, which concern technical and psychological issues that prevent to obtain optimal performances with every subject.

Unfortunately, communication is just one of the problems in ALS patients. In fact a variable range of cognitive impairments in moderate to severe stages of the disease is reported in many studies. The problem arises from the difficulties in performing a standard neuropsychological battery, which is generally made by tests and self-reports. Clinical evidence shows that it is sometimes almost impossible to perform a correct patients’ evaluation with such instruments.

In our opinion, BCI could represent an improvement of such situation. The development of a specific neuropsychological battery, adapted to get answers from ALS patients through the BCI, could represent a challenge for researchers and a great chance for ALS patients.

On the other hand, the use of BCI for AAC with these patients shows several limitations. These obstacles are partly due to technical issues, such as the transportation of the equipment and the recording quality in ecological settings different from the laboratory or the electrical artifacts that can alter signals. Other issues to be considered are the fatigability of the patients and the degree of distress they can feel, especially during the training and the initial phases of the use of BCI. Furthermore, the presence of cognitive impairment should be taken into account to fully understand if the poor results on BCI are due to patients’ cognitive deficits in comprehension, attention, concentration, etc. These important issues will be discussed in the following sections.

## Brain-Computer Interface

As previously mentioned, a BCI is a communication system that enables the generation of a control signal from brain responses such as sensorimotor rhythms and evoked potentials; it bypasses motor output and conveys messages directly from the brain to a computer. Therefore, it constitutes a novel communication option for people with severe motor disabilities, such as ALS patients. These systems can use a variety of different electrophysiological signals.

This review summarizes the current state of P300-based BCI systems focusing on its application for ALS patients.

### Definition and essential features of a BCI system

**Figure 1:**
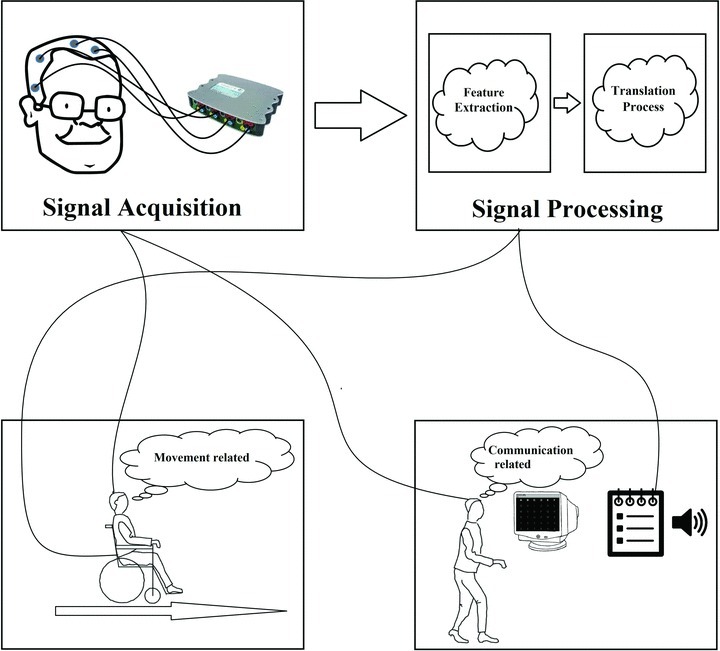
Schematic design and process of any BCI system.

BCI is a communication system that does not depend on the brain's normal output pathways of peripheral nerves and muscles (Fig. 1; Wolpaw et al. 2000); it is a technical interface between the human brain and a computer, that allows communication. Users explicitly manipulate their brain activity instead of using motor movements to produce signals that can be used to control computers or communication devices. As a matter of fact, a BCI system sends a message via brain activity to an external device, which performs the desired action. In order to successfully use a BCI, feedback and the following adaptation of brain activity are extremely important. Brain activity can be monitored by several methods. It is mainly based on the Electroencephalography (EEG) to measure, for example, event-related brain potential (ERPs), but other techniques are similarly available such as magnetoencephalography (MEG), positron emission tomography (PET), functional magnetic resonance imaging (fMRI), and functional near-infrared spectroscopy (fNIRS) (Wolpaw et al. 2000). However, PET, fMRI, and fNIRS are technically demanding and very expensive. Besides, they have a slow time resolution that does not allow rapid communication. Only EEG has a relatively short time constant, can be operated in many environments, and requires inexpensive devices, so it is the most practical and suitable method for BCI development.

### Learning a new skill

To successfully use BCIs, with the exception of the P300-based BCI, users have to learn to intentionally manipulate their brain signals. An approach for training users is called operant conditioning and provides users with continuous feedback as they try to control the interface (Tan and Nijholt 2010). The normal neuromuscular output pathways require feedback in order to successfully perform operations. A BCI that works as a replacement for these normal output channels also depends on feedback and on adaptation of brain activity based on the feedback. Thus, a successful BCI requires that the user develops a new skill, that is, the control of specific electrophysiological signals, and that the BCI turns this control into an output, which should correspond to the user's intent. As mentioned, a certain level of training is required for this dual adaptation between the computer and the user. One of the main problems related to the use of BCI with ALS patients is the fatigue for the sustained attention that is required to learn how to regulate the brain activity. Several studies of BCI with ALS patients, in fact, show that they may not be able to learn the skill to regulate brain activity because they are too weak to tolerate long-term training with focused attention (Kubler et al. 1999; Hill et al. 2006). Bai et al. developed a user-friendly BCI which requires minimal training and less mental effort for ALS patients. With this BCI ALS patients could achieve a good accuracy in a BCI paradigm associated with human natural motor behavior.

### Dependent and independent BCIs

There are two different classes of BCIs: dependent and independent. A dependent BCI does not use the brain's normal output pathways to carry the message, but activity in these pathways is needed to generate the brain activity (Wolpaw et al. 2002). For example, a dependent BCI uses a matrix of letters that the subject selects by looking directly at it, so that by recording the visual evoked potential (VEP) from the scalp over the visual cortex it is possible to determine gaze direction (Sutter 1992). In this case, the brain's output channel is EEG but this signal depends on gaze direction and therefore on extraocular muscles and cranial nerves that activate them.

In contrast, an independent BCI does not depend in any way on the brain's normal output pathways, because the message is not conveyed by peripheral nerves and muscles and the activity in these pathways is not needed to generate the brain activity. For example, an independent BCI uses a matrix of letters that the subject selects by producing a P300-evoked potential when certain letters flash (Donchin et al. 2000). The brain's output channel is a certain signal in the EEG and the generation of this signal does not depend on the orientation of the eyes, but on the user's intent (Sutton et al. 1965; Donchin 1981; Fabiani et al. 1987). This second group of BCIs seems to be more useful for ALS patients, who show damage in the output ways of peripheral nerves and muscles.

### The elements of a BCI

There are four essential parts of a BCI: (1) information input (i.e., recorded brain activity from the user), (2) signal processing (i.e., the components that translate raw information into output), (3) output (i.e., the commands administered by the BCI system), and (4) operating protocol that determines the timing of operation. These elements interact in order to produce the user's intention.

1. Signal acquisition is the measurement of the electrophysiological activity of the brain. This measurement is usually recorded via electrodes that can be either noninvasive (e.g., EEG) or invasive (i.e., intracortical). Moreover, BCIs can be categorized by whether they use evoked (e.g., EEG signals elicited by flashing letters) or spontaneous (e.g., EEG rhythms over cortex) inputs. Evoked inputs are generated by sensory stimulation provided by the BCI, while spontaneous inputs do not depend on such stimulation. The most common type of input is EEG recorded from the scalp (Vidal 1977; Farwell and Donchin 1988; Pfurtscheller et al. 2000; Freeman et al. 2003). In this first part of BCI systems, the input is acquired by the electrodes, then amplified, digitalized, and sent to the BCI system for further analysis.
2. Signal processing is the procedure to extract specific signal features that reflect the user's intent. In the signal processing stage, feature extraction process is carried out and the features are then converted, through translation algorithms, into commands that can operate and control devices (for a review see Norani et al. 2010).
3. The output device is usually a computer screen and the output is the selection of targets (letters or icons) presented on it, performed by the BCI (see, for example, Farwell and Donchin 1988; Wolpaw et al. 1991; Perelmouter et al. 1999; Pfurtscheller et al. 2000). These targets are flashed or indicated in various ways. Other BCIs output includes moving a cursor on the screen, controlling a robotic arm, or controlling some other physiological process.
4. The operating protocol guides the BCI operations. It defines how the system is turned on and off, what kind of feedback is provided to the user, the sequence and the speed of interactions between user and system, and the speed with which the system implements commands (Wolpaw et al. 2002; Leuthardt et al. 2009). In most research protocols, the investigator sets these parameters and the users do not have on/off control, they just have to achieve very limited goals and tasks. However, in real life the user must be able to do these things by her- or himself and such differences can make the shift from research to application difficult.

### Brain activity detection through EEG

A BCI system needs an input from the user's brain and these signals are converted in external operations; for this reason, brain signals have to be detected. EEG usually uses small electrodes placed directly on the scalp at standardized positions. When a neuron is activated, a local current flow is produced and weak potential differences (5–100 μV) between electrodes are measured. A large population of active neurons must be involved to generate electrical activity that can be detected with EEG over the scalp (Srinivasan et al. 1998). The electrodes record brain activity that is converted into digital signals and a sequence of steps translate this signals into commands.

A limiting issue with EEG recording is its low spatial resolution, ranging between 2 and 3 cm. Moreover, EEG is deduced from apical dendrites of cortical pyramidal cells (Teplan 2002), thus activity of deeper structures can only be studied indirectly. Because of the fluid, bone, and skin that separate the electrodes from the electrical activity, signals tend to be smoothed and noisy. This makes it difficult to locate the exact source of the oscillation. Nevertheless, EEG-based BCI have been shown to support a high performance, EEG is the predominant technology in BCI studies and most of clinical applications of BCI in patients with severe motor disorders have been demonstrated using EEG (e.g., Kubler et al. 2005; Vaughan et al. 2006; Nijboer et al. 2008). The changes in power of four frequency bands are used as control signals for BCI systems: delta (1–3 Hz), theta (4–7 Hz), alpha (8–12 Hz), and beta (13–30 Hz).

### Four groups of electrophysiological signals in a BCI system

As mentioned above, different noninvasive electrophysiological signals can be used as input for BCI systems. Therefore, BCIs can be classified into four groups based on the electrophysiological signals they use.

1. Visual evoked potentials (VEP)
  VEPs are evoked electrophysiological potential that can be recorded throughout the visual system; they are extracted, using signal averaging, from the electroencephalographic activity recorded at the scalp. VEPs are elicited by visual stimuli such as flashes of light or flickering illumination presented on a screen. Users are presented with a panel with different items and they have to fix their gaze on the item they want to select. The items on the screen are activated sequentially to elicit a visual evoked potential. BCI detects the VEP elicited by the stimulus where the subject looked at and the waveform of the VEPs depends upon the temporal frequency of the stimulus. In patients with neurological disease such as ALS, Sutter (Sutter 1992), for example, described communication problem with BCI due to artifacts caused by fasciculations. Moreover, the VEP-based BCI requires that the user is able to control gaze direction (Kubler et al. 2001b) and this is a problematic issue for patients with completely locked-in syndrome. This kind of communication system is categorized as dependent BCI, because it depends on muscular control of gaze direction.
2. Slow cortical potentials (SCP)
  SCPs are slow voltage changes generated in the cortex. Users can learn to control SCPs, although it requires a long training. Several studies showed that SCPs originating from central and frontal regions could be brought under voluntary operant control after training (Lutzenberger et al. 1993) and the importance of the anterior brain systems for the control of these functions has been further confirmed. As a matter of fact, patients with prefrontal dysfunction show extreme difficulties in learning SCP control, even if other cognitive functions are preserved (Lutzenberger et al. 1980; Birbaumer et al. 1986; Schneider et al. 1992). It is suggested that also patient with ALS are unable to voluntarily control local cortical excitation, because of the involvement of motor and premotor cortical systems in this disease.
3. Mu rhythm (sensorymotor rhythms SMR)
  Mu rhythm refers to 8–12 Hz EEG activity that can be recorded over primary motor and somatosensory cortex when awake subjects are not engaged in processing sensory input or producing motor output (Niedermeyer 2004). It is usually accompanied by 18–26 Hz beta-rhythms. SMR are associated with cortical areas most directly connected to the brain's motor output pathways. Movement or preparation of movement is associated with a decrease in mu and beta rhythms, labeled “event-related desynchronization” (ERD), while relaxation is accompanied by a rhythm increase or “event-related synchronization” (ERS) (Pfurtscheller 1999; Pfurtscheller et al. 2000). Notably, these rhythm changes occur also with motor imagery (i.e., mental representation of a movement) and do not require effective movement (Pfurtscheller and Neuper 1997; McFarland et al. 2000). Therefore, they may be used in independent BCI systems, which can be successfully adopted by paralyzed patients.
4. P300
  P300 evoked potentials are the best studied ERPs and they can be used as control signal in BCI systems. In the next paragraph, P300-based BCI will be extensively treated.

### P300-based BCI systems

#### P300 event-related potentials

**Figure 2:**
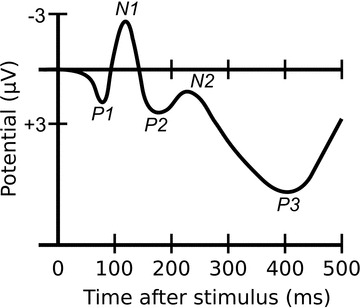
A schematic representation of some components of an ERP.

**Figure 3:**
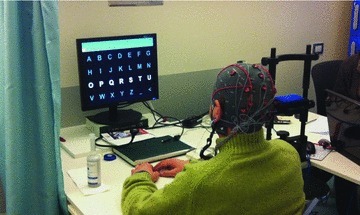
A P300 BCI session.

The P300 event-related potential is one possible EEG-based BCI control signal. These signals include both spontaneous electrical activity of the cerebral network and the cortical response to external or internal events. Event-related potentials are defined as brain activity that is elicited in response to events (Figs. 2 and 3; Donchin et al. 2000). ERPs can be distinguished in exogenous and endogenous. The former are the result of early and automatic processing of stimuli, whereas the latter correspond to later and more conscious processing of stimuli (Kubler et al. 2001b). Conscious processing occurs only after 100 msec, when the visual signal is under way toward extrastriate areas and areas in the parietal and temporal cortex, while unconscious processing starts in the first 100 msec and still occurs after this latency. Since endogenous ERPs depend on subjects’ attention to contextual stimuli and intentionality, they seem more suitable to be used in BCIs, with respect to exogenous ones.

The P300 is a deflection in the EEG that occurs 200–700 msec after stimulus onset and is typically recorded over central-parietal scalp locations (Fabiani et al. 1987).

The response is evoked by attention to rare or surprising, task-relevant stimuli in a random series of stimulus events (Fabiani et al. 1987), by mean of a simple discrimination task. In this “oddball paradigm” (Farwell and Donchin 1988), two stimuli are presented in a random order such as one of them occurs relatively infrequently, that is the oddball. The subjects are required to discriminate the infrequent target stimulus from the frequent standard stimulus, by responding covertly or overtly to the target. Subjects can be instructed to mentally count the target stimuli or to provide an overt response, such as pressing a button or make a finger movement when a target stimulus is detected. Events from the rare category elicit the P300 component of the ERP. Besides, a modification of the oddball task is the three-stimulus paradigm, in which infrequent distractor stimuli are inserted into the sequence of target and standard stimuli. In this case, a novelty P300 can be produced, named P3a, which is an early peak, large over the frontal and central areas and is thought to reflect frontal lobe function. P3a can be elicited also for typical, rather than novel, stimuli, when the perceptual distinctiveness between the target and the standard stimulus is quite difficult and the distractor stimulus is not novel, but highly discrepant. On the contrary, P300 arising from the target stimulus detection is a later peak with a large parietal amplitude, and has been called P3b, which is synonymous with P300 (Polich 2004). While P3a is produced when a demanding stimulus automatically drives frontal lobe mediated attention, P3b is produced when attentional resources are intentionally allocated for stimulus classification. From a neuroanatomical point of view, the P3a is thought to reflect activity of the anterior cingulate gyrus when new stimuli are processed into working memory. The P3b is thought to reflect subsequent activation of the hippocampal formation when frontal lobe mechanisms interact with the temporal/parietal lobe connection (Polich 2007; Verleger 2008). High task difficulty increases focal attention and enhances P3a amplitude by constraining other memory operations that reduce P3b amplitude and increase P3b latency (Hagen et al. 2006). Most P300 clinical studies have employed the P3b subcomponent.

The P300 is a slow wave oscillation associated with behavioral relevant and attention processes; it can be considered as an index of the amount of central nervous system (CNS) activity related to incoming information processing. Besides, it is thought to detect brain activity related to working memory when the neural representation of the stimulus environment changes with new sensory input. For these reasons, it has been used as a measure of cognitive functions in both healthy subjects and patients. The P300 component is measured by assessing its amplitude (size) and latency (timing). While the amplitude consists of the voltage difference between a prestimulus baseline and the largest positive-going peak of the ERP waveform within a latency window, latency is defined as the time from stimulus onset to the point of maximum positive amplitude within the latency window (Polich 2004).

In particular, P300 latency reflects stimulus classification speed, with shorter latency associated to better cognitive performances in attentional and immediate memory tasks. P300 measures are affected in neurological and psychiatric disease. Moreover, latency is not dependent on overt behavioral response and reaction times, so that it can be used as a motor-free measure of cognitive function. In order to distinguish the P300 from the background activity, generally dozens or hundreds ERPs are generated and averaged, so that the noise influence can be cancelled.

The amplitude of P300 is influenced by several factors: the target probability of appearance, the amount of time passed between the presentation of two stimuli, habituation effects, attentional and motivational issues, and the task difficulty (Gonsalvez and Polich 2002; Hoffmann et al. 2008; Kleih et al. 2010). In particular, the amplitude of P300 can be decreased in presence of highly probable events, with the probability for the target stimulus set to values around 10%. Besides, the shorter is the amount of time between two stimuli, the lower becomes the P300 amplitude. The P300 amplitude can also be decreased by habituation effects, which can appear when repeatedly presenting the same item, but only when short interblock intervals and many trial blocks are used (Polich and McIsaac 1994). Finally, fatigue effects, with a reduction in attentional capabilities, and a high task complexity can cause a reduction in P300 amplitude.

Also biological factors influence P300 amplitude and latency. Polich (2004) distinguished among natural factors (such as circadian, i.e., body temperature and heart rate, ultradian, seasonal, and menstrual cycles), environmentally induced factors (exercise, fatigue, drugs, and alcohol assumption), together with constitutional (age, gender, handedness), and genetic components. Biologic ERP effects can be reduced by ensuring that subjects are assessed similarly with respect to most of these variables.

#### Auditory, visual, and tactile P300-based BCIs

P300 used in BCIs can involve visual, auditory, or tactile stimuli presentation. In the field of BCI systems development, an important issue is to determine if a BCI device can work effectively using different presentation modalities, since possible users may have auditory or visual deficiencies. Previous research has shown that both auditory and visual oddball tasks elicit large P300 responses (Squires et al. 1977; Duncan-Johnson and Donchin 1982; Fabiani et al. 1987). In addition, McDonald et al.(2000) and Teder-Salejarvi et al. (2002) reported higher accuracy and larger ERP amplitude when auditory and visual stimuli were presented simultaneously, than when either modality was presented by itself.

Farewell and Donchin (1988) first used P300 to select items displayed on a computer monitor, by presenting participants with a 6 × 6 matrix, with each of the 36 cells containing one character. Participants were asked to pay attention to one of those cells, while the matrix rows and columns flashed in random order. In one trial of 12 possible flashed lines (six rows and six columns), the target cell flashes only twice: once in a column and once in a row. These two rare events typically elicit a P300 response. This example of oddball paradigm has been employed in order to build a P300 speller system, allowing users to communicate by mean of EEG recording.

A main issue of visual P300 is the use in subjects that suffer from visual impairments. In fact, users are required to fixate the matrix cell on the screen and to concentrate on itFor such reason, a preserved visual attention is supposed to be necessary in order to use P300 BCI. Treder and Blankertz (2010) investigated if a good performance at BCI depends on eye movements control (i.e., overt attention) or whether it is also possible with targets in the visual periphery (covert attention). They found that ERP-based BCI can be driven in both modes of attention, but the performance was significantly better for overt attention. The authors suggest the importance of developing innovative spellers that are reliably based on peripheral vision, since most of ALS patients show impaired eye movements. Also Brunner et al. (2010) explored this issue and found that the accuracy of P300 speller is affected by gaze direction, so its clinical applicability in ALS patients with impaired gaze may be limited. In such cases, auditory stimuli could be more suitable. The auditory version of the oddball tasks uses two different tones and an interstimuli interval of a few seconds, with the target stimulus occurring less frequently than the standard stimulus. As in the classic visual paradigm, the subject is required to distinguish between the two tones by responding to the target with a covert or overt response.

Only few studies have employed auditory oddball to elicit particular event-related potentials with P300 BCIs systems (see, for example, Hill et al. 2006; Sellers and Donchin 2006; Furdea et al. 2009; Klobassa et al. 2009). Hill et al. (2006) used a paradigm of two simultaneous pure tone streams, in which subjects focused their attention on one of the streams and ignored the other to make a binary decision. Sellers and Donchin (2006) tested healthy volunteers and ALS patients with a P300-based BCI. The words were presented visually, auditorily, or in both modalities. The authors were able to show that although the visual and visual plus auditory modality reached higher accuracy levels, a P300-based BCI using the auditory modality is feasible for both healthy and disabled subjects. However, the speed of the system is reduced, since spoken words were used.

The major limitation of some of these paradigms is that they provide no more than two to four alternative choices per trial. An auditory spelling system was presented by Furdea et al. (2009), which realized a multichoice auditory BCI by a 5 × 5 matrix of spoken numbers. Each character's position in the matrix was coded by two auditorily presented number words: one corresponding to the row and one corresponding to the column. To select a particular target character, the participant had to attend to the two target stimuli representing the coordinates of the character in the matrix. The subjects were instructed to first select the row number and then the column number containing the target letter. The authors found lower accuracy in the auditory modality than in the visual modality. Klobassa et al. (2009) designed a paradigm that uses auditory stimuli to operate a 6 × 6 P300 speller, thereby increasing the number of choices per trial to 36. Even if they found a higher accuracy with respect to previous studies using auditory BCIs, however, the speed and accuracy of the auditory speller was still lower than that of the visual version. In fact, average accuracy for the 6 × 6 36-item matrix for the visual P300 speller is typically 80–90% (e.g., Krusienski et al. 2006; Sellers et al. 2006), whereas in this study the mean online accuracy of the auditory P300 speller for the last sessions was about 66%.

BCI based on EEG responses to vibrotactile stimuli has the advantage of not requiring the presence of preserved visual or auditory system and of being potentially unnoticeable to other people. Moreover, they can be used in navigational applications, since a correspondence between the tactile stimulation and the spatial information is present.

Brower and van Erp (2010) investigated the feasibility of a tactile P300-BCI. Participants were asked to attend to the vibrations of a target, embedded within a stream of distracters. The number of targets was two, four, or six. The authors did not find a difference in Step-Wise Linear Discriminate Analysis (SWLDA) classification performance between the different numbers of tactors. They demonstrated the feasibility of a tactile P300 BCI and also proved that the stimulus onset asynchrony (SOA) for an optimum performance was close to the conventional SOA of visual P300 BCIs.

### P300-based BCIs: advantages and challenges

P300-based BCIs are independent BCI systems, since the generation of P300 does not depend on the exact orientation of the eyes and on the activity of peripheral nerves and muscles, but it mainly depends on the user's intent to pay attention to one stimulus. With the limitations described below, its use is possible with patients suffering from impairment in oculomotor dysfunctions, such as ALS and locked-in patients.

Besides, a P300-based BCI does not require initial user training in order to generate a P300 in response to the desired target. In a recent study, Guger et al. (2009) proved that the P300-based BCI can achieve high accuracy after only 5 min of training. In such study, 72.8% of the subjects reached 100% accuracy with a row-column paradigm speller. Interestingly, they found that the system was more accurate for people who slept less the previous night, while no significant differences were observed with regard to gender, level of education, working duration, and cigarette and coffee consumption. These results overcome those obtained in a previous study by Guger et al. (2003), where they tested a motor imagery-based BCI system and found that, after 20–30 min (two sessions) of training, about 93% of the subjects were able to achieve classification accuracy above 60%. These findings highlights that P300-based BCIs are a far more practical choice than SMR-based BCIs. In P300-BCIs, P300 ERPs from several trials are averaged, in order to improve accuracy and reduce noise. The classifier discriminates which stimuli elicit a robust P300. If none of the stimuli provoke an ERP different from other ERPs, this indicates that it is not possible to use P300 for communicating. Such phenomenon has been observed across different BCI approaches, with 20% of subjects being not proficient in using BCI, and it has been called “BCI illiteracy” (Kubler and Muller 2007). The main explanation of such phenomenon is that not every person can generate the brain activity necessary to control a specific BCI. In fact, even if all people's brain shares (more or less) the same functional properties and subdivisions, some differences in brain structure can be present. For example, some users produce P300 evoked-related potential not detectable at the level of the scalp, so that EEG cannot be effectively performed. In particular, it has been observed that 10% of healthy subjects do not produce a robust P300.

Some issues must be considered while planning to use a P300-BCI system. Two important criteria in order to evaluate the feasibility of a BCI system are the speed and the accuracy (Kubler et al. 2001a). The former is related to the fact that the more rapidly a BCI can be controlled, the more amount of information can be produced by the user, and the faster communication is possible. Obviously, compared to speech, the communication rate is reduced with BCI, but a limit presumably exists, below which the communication rate of a BCI should not fall.

Accuracy is the percentage of correct selections per time intervals. It represents a relevant issue, since a wrong selection could turn into an error in communication, with both practical and psychological consequences for the user. In order to avoid it, the BCI system must be equipped with options that allow a user to correct wrong selections. A balance between speed and accuracy should be identified. Besides, accuracy is diminished also by the close temporal proximity of multiple target stimulus presentation. More specifically, the close temporal proximity of target stimuli leads to severely diminished accuracy (Martens et al. 2009; Salvaris and Sepulveda 2009; Citi et al. 2010). Although this phenomenon may have been detected early on (Serby et al. 2005), recently a more concerted effort has been observed in the literature in order to try to overcome such limitation.

Moreover, technical challenges are related to the recording quality in environment different from the laboratory setting, such as the user home, when different sources of noise can disturb the EEG recording (Sellers et al. 2006). Besides, the patient respirators may introduce electrical or mechanical artifacts. In the end, perceptual and cognitive abilities, in particular the capacity to pay selective and sustained attention to the target stimuli must be considered when employing P300 with neurological patients. It is necessary to determine whether or not a user is able not only to see the computer display, but also to focus on a particular stimulus on the display. Furthermore, since using a P300-BCI requires attention and concentration and interference may occur between counting the number of flashes of the matrix cell and simultaneously concentrating on the characters to be selected, the user should not be distracted. Therefore, it may be difficult to use P300 in everyday life. Some pilot studies have shown that some patients may not be able to learn the necessary skills for proper and effective use of the P300-based BCI, due to an excessive distractibility and an incapacity to tolerate a long-term training (Kubler et al. 1999; Hill et al. 2006).

Other issues that may be problematic, especially during the initial stages of training with the BCI, are the boredom and the frustration that are sometimes reported by the patients themselves. However, a recent study shows that motivational aspects are positively correlated with performance related to the BCI, by suggesting that highly motivated patients can get good results from the use of BCI as a communication tool; besides, some benefits with regard to patients psychological well-being seem to arise from satisfaction for the obtained results (Martens et al. 2009; Kleih et al. 2010).

When planning to use P300 as an AAC device, it should be taken into account that the use of such technology is not always easy to understand for the patient and often a training is needed, which should involve both the patient and his/her family. Besides, the AAC system should be developed according to the communicative needs of the specific person, and the tools provided must be flexible and adapt to the changes which may occur in the person needs (psychological, emotional, and social) and abilities. In fact, most of the patients who use BCI devices show some degree of cognitive impairment, which may has negative effects on the performances. Thus, it is compelling to extensively assess the presence of cognitive deficits and this is particularly relevant for ALS patients according to the most recent findings.

## Cognitive Impairment in ALS

Although ALS is traditionally described as a pure motor disease, evidence has accumulated that ALS is a multisystem disease that also involve a range of cognitive deficits in most patients, with a small proportion (5–15%) meeting criteria for frontotemporal dementia (FTD). Frequency, severity and types of cognitive impairments in ALS vary widely. The reason lies partly in the source of patients and in the different methods used to assess cognition in the different series of ALS patients. Early reports suggested that the prevalence of cognitive impairment was about 1–4% (Brownell et al. 1970; Jokelainen 1977; Eisen and Krieger 1993; Strong et al. 1996), but one of the largest study so far found a significant cognitive impairment in 36% of nondemented patients (Massman et al. 1996). In more recent studies, the occurrence of cognitive deficits in ALS without dementia has been reported in up to 50% of patients (Abe et al. 1997; Lomen-Hoerth et al. 2003; Phukan et al. 2011). Although cognitive assessment in patients with ALS is difficult due to the severe physical disabilities related to the disease itself, the most consistently reported cognitive changes regard frontal executive functions, that is, verbal fluency, mental flexibility, attention, working memory, planning, and abstract reasoning. Dysfunctions in memory and language are also present, but to a lesser degree.

Verbal fluency has been found to be impaired in the majority of cognitive studies in ALS (Gallassi et al. 1989; Ludolph et al. 1992; Kew et al. 1993; Abe et al. 1997; Abrahams et al. 2000, 2005b; Lomen-Hoerth et al. 2003). Both letter and category fluency seem to be disturbed and this simultaneous impairment reflects dysfunction in components of the executive system. Abrahams et al. (2000) related the impairment on tests of intrinsic response generation, that is, Written Verbal Fluency Test, Category Fluency Test, and Design Fluency Test, to a higher order dysfunction, implicating deficits in the central executive component of working memory; these deficiencies do not depend on an impairment in primary linguistic ability. Letter fluency deficits in ALS have been shown to be independent of motor disability and speech weakness using a written version, which includes a motor control condition and correction for motor speed (Abrahams et al. 1997, 2000).

Difficulties with concept formation and mental flexibility measured by the Wisconsin Card Sorting Test have also been reported in many studies (David and Gillham 1986; Massman et al. 1996; Abrahams et al. 1997; Evdokimidis et al. 2002). However, according to Phukan et al. (2007), deficits in this test are less reliably found than in other tests like in those for verbal fluency in patients with ALS. Accordingly, findings of impaired performance on WCST have not been confirmed in several studies (Ludolph et al. 1992; Kew et al. 1993; Talbot et al. 1995).

Impairments in the attentional system are often associated with damage of the frontal lobes. Attention deficits have been described in ALS, especially with concern to selective and divided attention (Vieregge et al. 1999; Schreiber et al. 2005; Pinkhardt et al. 2008). Problems with concentration and distractibility have been described for the Stroop Test; Evdokimidis et al. (2002) correlated the difficulties in Wisconsin Card Sorting Test and Stroop Test with distractibility factor scores for an anti-saccade ocular motor paradigm, which represents another valid test of frontal lobe function, that allows to avoid motor and verbal responses.

Patients with ALS show cognitive deficits also in other areas than executive function, but the evidence is less consistent. For example, there is no agreement about memory decline: several studies have reported impairment in short-term memory (Gallassi et al. 1989; Kew et al. 1993; Hanagasi et al. 2002), while deficits in delayed recall are variable, suggesting a disorder in the encoding of information rather than in the speed of forgetting. Mantovan et al. (2003) detected a poor primacy effect, that is indicative of a long-term memory deficit, and suggested that poor performance on memory tests may be indicative of a failure to generate stable long-memory traces at encoding rather than a failure in memory retrieval. However, there is no agreement in the interpretation of memory failures in terms of frontal lobe dysfunction: impairments in delayed recall (of three words and a short story) were assumed by Iwasaki et al. (1990) as an effect of medial temporal lobe pathology, on the basis of the role of the medial temporal lobes in recall of learned information.

Language abnormalities have also been described and they included impoverished verbal output (Strong et al. 1999; Bak and Hodges 2004), deficits in confrontation and objects naming (Massman et al. 1996; Strong et al. 1999; Bak and Hodges 2004; Abrahams et al. 2005), difficulties in syntactic comprehension (Rakowicz and Hodges 1998), and paraphasias (Rakowicz and Hodges 1998; Strong et al. 1999). However, some studies have found no naming deficits in ALS patients (Kew et al. 1993; Abrahams et al. 2000), so the existence of a true aphasic disorder is still a matter of debate. Moreover, the difficulty in distinguishing articulatory from linguistic disturbances complicates the interpretation of the aphasic-like symptoms. Most authors ascribed the language impairment to executive deficits rather than to aphasia; poor verbal fluency or errors in sentences comprehension in the absence of symptoms of expressive or receptive language impairment, for example, result from a higher order executive deficit rather than primary linguistic abilities (Talbot et al. 1995; Abrahams et al. 2000). On the contrary, other studies interpreted difficulties in comprehension as an evidence of aphasia (Doran et al. 1995). According to Tsermentseli et al. (2012), however, there is currently insufficient evidence to support the idea that language deterioration is related to executive dysfunction rather than to an aphasic syndrome.

Emotional processing and social cognition have also been investigated in ALS. Papps et al. (2005) found a failure to show the normative pattern of enhanced recognition memory for emotional words in ALS patients. In addition, one study showed that in early stages of the disease emotional responses of ALS patients tend to be altered toward positive valence and toward a more balanced arousal state: they express more positive verbal emotional judgements and rate exciting pictures as less arousing and exciting than controls (Lule et al. 2005). In a group of nondemented ALS patients Girardi et al. have found a deficit of Theory of Mind, that is, an impairment in inferring the mental state of another on the basis of a simple social cue, that is over and above the presence of executive dysfunctions and suggests a profile of cognitive and behavioral dysfunction indicative of a subclinical FTD syndrome.

Behavior impairment is now recognized as another typical feature of ALS and cognitive and behavioral impairments can coexist in approximately 25% of ALS patients (Newsom-Davis et al. 1999; Murphy et al. 2007). Up to 63% of patients are apathetic, irritable, inflexible, restless, and disinhibited (Lomen-Hoerth et al. 2003; Murphy et al. 2007; Phukan et al. 2007). Emotional lability, that is, the patho-logical occurrence of sudden episode of laughing or crying, has been estimated in 10–20% of ALS patients (Newsom-Davis et al. 1999). However, those episodes are not necessarily in line with the emotional state of the patient. The prevalence of ALSbi, that is, behavioral impairment that does not meet diagnostic criteria for FTD, varies depending on methodology and diagnostic criteria. One feature which is consistent across many studies is the presence of apathy. Grossman et al. (2007) used the FrSBe (Frontal System Behavioral Scale) to assess changes in apathy, disinhibition, and executive dysfunction in ALS patients; results showed a high incidence of behavioral changes, particularly regarding apathy (55%), and emphasized the usefulness of the scale for detecting behavioral functioning in these patients. Bulbar-onset disease was significantly related to apathy ratings indicating that patients with bulbar-onset ALS are more likely to develop behavioral symptoms than those with limb-onset disease. This finding is consistent with previous reports demonstrating that cognitive dysfunctions are more common in individuals with bulbar-onset ALS (Abrahams et al. 1997; Strong et al. 1999; Lomen-Hoerth et al. 2003; Schreiber et al. 2005; Ogawa et al. 2009). Although increased cognitive impairment in bulbar-onset patients is frequently described, other studies have failed in finding a link between bulbar-onset and cognitive decline (Kew et al. 1993; Mantovan et al. 2003; Ringholz et al. 2005; Rippon et al. 2006).

In conclusion, these composite studies show that a significant subgroup of ALS patients exhibit cognitive deficits affecting frontal lobe functioning, specifically in planning, attention, and verbal, and nonverbal fluency. There is also minor involvement in memory and language skills, which could be due in part to frontal dysfunction. The level of abnormality ranges from overt dementia, meeting criteria for FTD, to subtle impairments detected only by neuropsychological testing. The neuroimaging studies in nondemented ALS patients strongly indicate an organic basis to the frontal deficits detected on neuropsychological testing and a task force to further detect nonmotor changes in ALS has been created (Tsermentseli et al. 2012).

### Longitudinal studies

With regard to the progression of the cognitive decline in ALS, the current opinion is that the cognitive impairment slowly declines over the course of the disease. Strong et al. (1999) found a progression over time of the cognitive deficits across several domains, including working memory, problem solving, mental flexibility, recognition memory for words and faces, and visual-perceptual skills in five patients with bulbar-onset ALS, while limb-onset ALS patients showed no decline at the six months follow-up. A MR spectroscopy following the neuropsychological testing demonstrated a significant neuronal loss in the anterior cingulate gyrus in bulbar patients that was evident early in the course of cognitive impairment and correlated with the appearance of impaired cognition. Another longitudinal study noted that cognitive deficits were present at initial testing and, after the early decline, seemed to remain stable over time in contrast to motor decline; in addition, bulbar-onset patients performed worst in many neuropsychological tests than spinal-onset ones and this subgroup difference increased on follow-up (Schreiber et al. 2005). These findings were replicated by another longitudinal study (Abrahams et al. 2005) in which selective deficits in spoken and written verbal fluency did not show deterioration over a six months period in a group of nondemented ALS patients. In a study by Robinson et al. (2006), no significant and meaningful between-group and within-group differences in cognitive function were found over time. Individual analyses, however, showed that seven of 19 ALS patients developed abnormal cognitive performances especially in tests that assess short-term recognition memory, hypothesis generation, working memory, verbal learning, and verbal long-term recognition memory over a six-month period.

Overall, most studies demonstrate a slow progression of cognitive symptoms in ALS relative to motor decline and show that these cognitive deficits are present early in the course of the disease.

### Overlapping between ALS and FTD

Although ALS and FTD are two different entities, it is now clear that these disorders are neurodegenerative conditions with overlapping clinical and neuropathological features. The overlap is further confirmed by the presence of the ubiquitinated Tar DNA binding protein (TDP-43) inclusions both in FTD patients without tau pathology, as well as in sporadic and familial case of ALS (Neumann et al. 2006; Kwong et al. 2007). Moreover, as will be described in the next section, ALS patients show an impairment of cerebral regions beyond the motor system, including cortical areas typically involved in FTD and a proportion of ALS patients displays cognitive and behavioral changes that in some instance reach criteria for FTD Strong and Rosenfeld 2003; (Irwin et al. 2007). Strong et al. (2006) found a pattern of mental change that was indistinguishable from that of FTD in a group of patients with dementia and ALS. In a large study involving 279 ALS patients, 50% manifest cognitive impairment and 15% met criteria for FTD (Ringholz et al. 2005). Murphy et al. (2007) found a spectrum of frontal lobe dysfunction in half of the patients, with five of them (22%) meeting neary criteria for FTD. These studies support the hypothesis of a clinical continuum between ALS and FTD, according to which FTD is an integral component of ALS and can be expected in any ALS patients. The timescale of onset and the pattern of the cognitive symptoms in FTD/ALS are not clear, but reports have suggested that FTD reflects one end of the disease continuum. However, this argument is difficult to support when considering that some studies have found no meaningful progression of cognitive deficits over time. If cognitive deficit exists, these could affect an assessment in these patients; in general, there is always cognition needed as prerequisite to test cognition. More, BCI could also have problems when deficits exists. To overcome this circularity could be more effective to use a P300 BCI system. The good thing about P300 BCI is that the response itself does not need major load of cognition to perform a P300 ERP (P300 is used in unconscious patients as well and an oddball paradigm should nevertheless work). So if the production of the signal does not need any cognitive load than testing with cognition with BCI is just the same as testing cognition with paper and pencil which is a standardized procedure. The use of BCI in ALS patients with cognitive impairment has already been studied (Iversen et al. 2008a; Perego et al. 2011).

### Neuroimaging and psychophysiological evidences of extra-motor involvement in ALS

Neuropsychological data are supported by neuropathological evidence obtained in ALS with both conventional and modern neuroimaging approaches highlighting extra-motor cortical atrophy in such disease. Voxel-based morphometry (VBM) studies showed that regional gray matter (GM) loss is not confined to motor regions, but is extended to the frontal, temporal, parietal, and limbic regions (Grosskreutz et al. 2006; Turner et al. 2007). In particular, the frontal regions have been observed to have the most severe atrophy in patients with ALS and FTD. By employing VBM, Abrahams et al. (2005a) reported white matter (WM) reductions in the medial temporal lobe, anterior cingulate gyrus, and medial frontal lobes in a group of ALS patients with impaired verbal fluency scores. Among the modern structural neuroimaging methods, diffusion tensor imaging (DTI) has provided evidence of significant reduction of fractional anisotrophy (FA) not only in CST but also in extramotor regions, including frontal, temporal, parietal and occipital WM, corpus callosum, the hippocampal formation, and the insula (Sach et al. 2004; Sage et al. 2007; Senda et al. 2009; Lule et al. 2010). Functional neuroimaging has supported the clinical findings of frontal cortical involvement not only in patients with an ALS/dementia complex but also in patients with ALS and subclinical cognitive impairment. Abnormal activations extending beyond the sensorimotor cortex in ALS has been proved in PET and fMRI studies during motor execution tasks and verbal fluency tasks. In particular, a hypoactivation has been measured in dorsolateral prefrontal cortex (DLPFC) in both conditions (Kew et al. 1993; Stanton et al. 2007). Furthermore, hypoperfusion in the frontal cortex in ALS with or without cognitive deficits measured with PET (Ludolph et al. 1992) and fMRI (Tanaka et al. 1993) and association of reduced frontal executive function and reduced activity in frontoparietal areas measured with PET has been shown (Abrahams et al. 1996). Other functional imaging studies have provided further evidence for extra-motor involvement in ALS (Lule et al. 2007; Han and Ma 2010; Mohammadi et al. 2011). The combination of neuropsychological measures and multimodal neuroimaging approach seem promising in highlighting the cerebral mechanism underlying ALS cognitive deficits, identifying the differential role of GM and WM dysfunctions.

An important contribution in the study of extra-motor functions is represented by event-related potentials. Some studies have showed that some ALS patients produce less typical ERPs than healthy matched subjects (Paulus et al. 2002). A previous ERP study in patients with sporadic ALS found that P3a and P3b amplitudes of ALS patients were lower compared with controls, and P3a latencies were significantly longer (Hanagasi et al. 2002); ERP recordings in nondemented patients with sporadic ALS also showed prolonged N200 and P300 latencies compared to healthy controls (Gil et al. 1995). By employing neuropsychological measures, ERPs and clinical scales, Ogawa et al. (2009) studied a sample of patients with early stage sporadic ALS. They found that patients with the bulbar-onset type showed marked prolongation of P3 latency compared to patients with the limb-onset type and controls. Furthermore, correlation studies revealed that the relative bulbar functional rating scale correlated with prolonged P3 latency and low P3 amplitude. These results further suggested that patients with bulbar-onset ALS had consistently poorer cognitive test performance than those with limb-onset ALS (Schreiber et al. 2005). In addition, a significant correlation was found between the respiratory function tests and P3 amplitude, by suggesting that ventilatory impairment overrides cognitive impairment caused by the disease itself.

The described evidences with regard to the P300 component of the ERPs in ALS patients suggest the presence of an impairment of novelty detection mechanisms, which are associated with the dorsofrontal–orbitofrontal and anterior cingulate cortices. Such results confirm the dysfunction of the frontal network in ALS, according to neuropsychological, neuroimaging, neuropathological, and genetic evidences and with the hypothesis of an overlapping between ALS and FTD.

The discussed abnormalities in brain structures and functions and in psychophysiology observed in ALS, which turn into an impaired cognitive profile in a consistent proportion of patients, apparently represent a challenge for the use of P300 as an input signal in BCIs. However, some studies have investigated this issue, providing encouraging results against the hypothesis of a generalized “ALS illiteracy.” In particular, (Kübler and Birbaumer 2008) investigated the relationship between the level of motor and physical impairment and the ability to use brain computer interface, by comparing three different BCI systems (P300, SCP, and sensorimotor rhytms [SMRs]). They found no continuous decrement in BCI performance with physical decline, even if in completed locked in state (CLIS) no communication was possible. According to these evidences, the major challenge remains the use of BCI-based systems with CLIS patients, who have the greatest need for a BCI in order to communicate.

### Cognitive assessment of ALS and locked-in syndrome (LIS) patients through BCI-based AAC systems

BCIs have been studied with the primary motivation of providing assistive technologies for people with severe motor disabilities, particularly locked-in syndrome (LIS) caused by neurodegenerative disease such as ALS or by stroke. These patients are conscious and alert but they are unable to use their muscles and therefore can not communicate neither vocally nor by writing (Kubler et al. 2001b). In LIS, vertical eye movements and eye blinks are spared while in the complete LIS (CLIS) patient lose any control of the eye muscular response.

BCI usually requires a training that can be physically and emotionally very exhausting for patients, especially when they show some degree of cognitive impairment. Moreover, the possibility that some of them could fail to use BCI at all must be considered.

Nowadays, the evaluation of cognitive abilities in patients at the advanced stage of paralysis, such as ALS patients, still represents a challenge, due to the fact that all standard assessment tools for both verbal and nonverbal cognitive abilities involve a motor response. Besides, even tests relying on some form of rudimentary motor function such as blinking, nodding, or pointing (Anastasia and Urbina 1997), are not administrable to totally locked in patients.

Iversen et al. (2008b), aimed at assessing some cognitive functions in completely paralyzed ALS patients. Based on previous results showing that some late-stage ALS patients can learn to communicate with high accuracy using only their EEG (Kotchoubey et al. 1997; Pfurtscheller and Neuper 1997; Kubler et al. 2001b), they developed a slow-cortical potentials (SCP) EEG BCI.

In a first study (Iversen et al. 2008a), training was applied to two severely paralyzed ALS patients, during which the patients could learn to control certain components of their EEG in order to direct the movement of a visual symbol on a monitor. Following, a series of two-choice cognitive task were administered. For example, a noun and a verb were presented, one in each choice target, and the patients were given the verbal instruction to steer the cursor to the noun on each trial. Similarly, other tasks assessed basic abilities such as odd/even number discrimination and discrimination of larger/smaller numbers, with stimuli varying according to the level of complexity. Performance was also assessed using a matching-to-sample paradigm, which was used to examine the ability to discriminate numbers, letters, colors, and to perform simple calculations.

In a successive study, Iversen et al. (2008b) employed the same SCP-EEG control in order to administrate a conditional-associative learning task to a late-stage ALS patient, testing the ability to learn arbitrary associations among visual stimuli. In both studies, a good level of accuracy was observed in detecting patients performances, according to a within subjects experimental design. Patients were also able to understand the verbal instructions and to respond accordingly in the successive tasks.

However, this method owns some important limitation: first, it requires an extensive pretraining in order to learn to control EEG, which can take some weeks; second, the method cannot be used for tasks based on recall or where a choice must be made among more than two stimuli.

Differently from all other existing BCIs, in P300-based BCIs learning of self-regulation of the brain response and feedback is not necessary. Moreover, the short latency of the P300 allows much faster selection of letters than any other BCI system. On the other side, the use of P300 requires, as a precondition, an intact visual system, at least for the visual modality, which has been proved to be more reliable than the auditory one, and preserved ability to pay attention, which can represent a problem in some patients.

Despite the several advantages of P300-based BCIs, to date this approach has not been employed yet for the cognitive assessment of locked-in patients.

However, some recent studies have preliminarly investigated the possible use of different kind of BCI-based systems to administer neurocognitive tasks (Cipresso et al. 2011; Perego et al. 2011). This could represent a promising step toward the application of such approach to ALS patients.

To date P300 has been mainly used for communication purposes, while the few attempts performed in the cognitive assessment of those patients have employed other approaches. There is no apparent reason for this; probably, works showing promising results in training SCP modulation in neurofeedback experiments have lead to focus on this approach, as showed by previous described studies (Iversen et al. 2008a, b). However, the possibility to reduce the amount of time required for the users training represents a very important chance in order to extend the use of AAC also for cognitive assessment purposes. Then, the field of research about the development of cognitive tasks based on BCI for patients with motor disabilities is still at its infancy, and represents an interesting area to be developed.

## Discussion: AAC with BCI for ALS and Future Challenge

The aim of the AAC is to provide solutions to facilitate and increase interaction between the user and his environment, using the ensemble of knowledge, strategies, techniques, and technologies most suitable to meet the user's needs for communication. Thanks to the technology made available by the AAC, many patients with severe disabilities can use tools that allow them to communicate (Beukelman et al. 2007).

As previously described, communication difficulties are the most critical symptoms as perceived by ALS patients. Dysarthria makes verbal communication progressively difficult and ultimately impossible. The choice of appropriate alternative communication methods is crucial and it ranges from pen and paper and alphabet board as long as the patient is still able to use upper limbs, to electronic communication devices. Patients with very limited mobility who cannot manually use a mouse or a keyboard can take advantage of the use of gaze communication systems, which use eye tracking to allow communication. Such systems have a camera mounted at the bottom of the screen that “tracks” the eyes as they move across the screen. The viewer's precise gaze-point at an onscreen keyboard is detected and it allows the patient to spell a message for speech or text output. Software also allows to switch lights and appliances on and off and to dial telephone numbers. The systems are compatible with personal computers or AAC devices.

One of the most critical needs for people with severe motor disabilities to retain a good quality of life (QoL) is restoring the ability to communicate. Several studies showed that the reported QoL of patients with CLIS is quite good if they can communicate with caregivers and family (Perelmouter and Birbaumer 2000).

Over the past 20 years, several patients with ALS and LIS have been trained with BCI not only in research laboratories, but also in their home.

Birbaumer et al. (1999) first developed a communication system for completely paralyzed patients with ALS that employed SCP to drive an electronic spelling device. Two patients were trained to produce voluntarily positive and negative SCPs and were provided by visual feedback. After achieving at least 75% control, they began to use the spelling device, in which letters were presented on a screen. Patients selected a letter by progressively reducing letter strings containing the desired letter by creating SCPs after its appearance. Many other studies described BCI systems based on self-regulation of SCPs in patients with physical impairment, thus supporting that these patients are able to learn to control changes in their SCPs accurately in a sufficient way to operate an electronic spelling device (Kubler et al. 2001a, b; Wolpaw et al. 2002; Birbaumer et al. 2003). Although letter selection is slow—1 min for 1, 2 letters—it is reliable and precise enough to allow patients to communicate.

Other researchers did extensive experiments using BCI based on SMR rather than on SCP. Wolpaw and McFarland (2004) demonstrated that a SMR-based BCI provides subjects with spinal cord injuries with multidimensional control of a cursor movement on a computer screen that could be learned in a few sessions of training. The speed, accuracy, and learning performance were comparable to those of invasive BCIs. Kübler et al. (2005) showed that four patients with ALS learned to operate a BCI with rhythms recorded over sensorimotor cortex and suggested that this kind of BCI system could help ALS patients maintain a fairly good QoL.

To confirm these encouraging results, other studies have confronted patients with various types of BCI to provide them with a system that works best for each individual subject. In a study investigating the effects of psychological state and motivation on BCI performance in ALS, Nijboer et al. (2010) made a comparison between SMR- and P300-BCI paradigms in order to assess which paradigm could be used by most patients. A higher information transfer rate with the P300-BCI, than with the SMR-BCI was found. Moreover, this information transfer rate could be achieved by patients after only one session with P300-based BCI, whereas the use of SMR-BCI required more extensive training (10 training session). These results support the hypothesis that P300 BCI requires no learning, while SMR regulation is a skill that has to be learned by training. Therefore, P300-BCI seems to be superior to the SMR-BCI for quick and reliable communication.

Other studies evaluated the effectiveness of BCI systems that operate by detecting a P300 signal. Sellers and Donchin (2006) assessed its effects across three different stimulation modes, that is, auditory, visual, and both, using a paradigm based on a four-choice oddball. The words “yes,”“no,”“pass,” and “end” were presented one at time as auditory, visual or auditory, and visual stimuli. The subjects’ task was to count the number of times the target, that is, “yes” or “no,” was presented in a random sequence of the four choices. The authors demonstrated that these stimuli can be used as a P300-BCI control signal and they supported the effectiveness of P300-BCI with a population of ALS patient, although the sample size was small (*N*= 3). To extend these initial findings, Nijboer et al. (2008) evaluated the ability to use a P300-based matrix speller to communicate spontaneous words and phrases in a larger group of individuals with ALS. They also tested the stability of their BCI performance in repeated sessions over a prolonged period of time. In a two-phase study, subjects completed the first 10 copy-spelling sessions (Phase I) and then 10 free-spelling sessions (Phase II). The results showed that severely disabled patients can use a P300-based BCI for both cued and spontaneous text production and that performance does not degrade over weeks and months, considering that the amplitude and latency of the P300 remained stable for up to 40 weeks.

Recently, Silvoni et al. (2009) described results of training and one-year follow-up of brain communication in early and middle stage ALS patients using a P300-BCI. In addition, they investigated the relationship between acquired BCI-skill and the clinical status, including cognition and the degree of physical impairment. A four choice visual paradigm was employed and the subjects were asked to reach with a cursor one of four icons on a screen, representing basic needs (i.e., “I’m hungry,”“I’m sleepy,”“I need a doctor,”“I would like something to drink,” etc.). The comparison between BCI-skill of the training and follow-up protocols did not reveal any difference, corroborating the hypothesis that patients maintain their communication abilities even after a long period and even if the physical impairment progresses, although the small sample size (*N*= 5) limits this conclusion. No significant relationship was found between BCI skills and clinical status, including the cognitive abilities. The positive correlation between patient's age and some BCI skill parameters showed that age could influence the acquisition of the BCI skills. The older and the more in need for a BCI a patient is, the greater is his motivation to achieve control over a BCI communication tool. Similar results were also found by Kübler and Birbaumer (2008) in a meta-analysis of all reviewed publications, in which the authors concluded that there was no relationship between severity of the disease, physical decline, and BCI performance, except for completely locked-in patients, who were unable to learn to use a BCI. Therefore, this study supports the idea that a BCI system can be used in ALS patients at early and intermediate stage of the disease, before entering the locked-in phase.

The usefulness of BCI in CLIS still remains a matter of debate. Kübler and Birbaumer (2008) reported that the BCI technology has been unable “to restore basic communication (yes/no) in patients who were in the complete locked-in state at the beginning of the training.” However, CLIS patients showed ERP responses to one or more complex cognitive task, thus indicating partially intact processing stages in the CLIS despite a reduced general arousal (Hinterberger et al. 2005). Assuming intact processing modules and possible transfer of already learned BCI communication from basic eye movement control to LIS and CLIS, the question why patients who enter the CLIS before learning BCI use do not acquire control of their brain signals remains to be determined. However, in order to prevent failure in BCI use, Kübler and Birbaumer (2008) suggest that users should be entered in BCI training before the beginning of total locked-in phase.

As mentioned above, another available technology for communication purposes is the eye-tracker system. However, a main limitation of this system is the need of a preserved ocularmotor ability, in order to point with the gaze toward the target (letter or pictures) to be selected. Even if visual P300 requires the patient to perform ocular movements and fixation to some extent, several studies show that it can be employed also with ALS patients in the late stage of the disease; in fact, no continuous decrement has been observed in BCI performance with physical decline (Kübler and Birbaumer 2008).

## Conclusions

Some difficulties in the effective use of P300 BCIs can be observed in neurological patients; among them, persons suffering from ALS presenting specific cognitive profiles. A first problem when using P300 with such patients could be related to the duration length of the training phase; in fact, even if P300 usually does not require more than a calibration phase with healthy persons, this does not always apply for patients. When planning to use a P300 BCI system with neurological patients, a high degree of flexibility must be considered. Due to the increased level of fatigability showed by such patients, it could be necessary to perform the training for a longer time and to perform an adequate number of breaks. Some cognitive difficulties more specifically related to ALS syndrome, such as poor concentration, distractibility, and short-term memory difficulties, should be taken into account, in order to adequately plan and realize AAC sessions.

As we already discussed, cognitive assessment in ALS patients is quite difficult to be performed, due to the motor-verbal impairment and the impossibility to use the traditional paper and pencil tools. BCI has been recently investigated as an alternative method to administer cognitive tasks, and the collected evidence seems to be promising (see, for example, Iversen et al. 2008a; Cipresso et al. 2012). At the moment, P300 BCI has not yet been employed in order to realize an alternative mean to perform a cognitive assessment of ALS patients, even if it seems to offer some main advantages with respect to other BCI systems available as discussed above. These are mainly the short latency of the P300, allowing much faster selection of letters, and the lack of need of training in order to learn to self-regulate the brain response and feedback.

Starting from these considerations, a short protocol based on the use of P300 BCI could be created. In particular, some traditional neuropsychological tests could be modified to create computerized short versions that could be easily adapted to BCI administration in a secondary step. Before administering such protocol to ALS patients, it should be necessary to obtain normative data from healthy population. So, a control group representative of the widest sample of patients, with regard to age and educational level, should be recruited and administered with the complete battery. Basing on these data, cognitive profile of ALS patients could be depicted. It is important to identify different strategies that allow a flexible and dynamic use of this complex approach with those patients who show cognitive impairments; for example, it could be useful to increase stimulus duration or its size, in order to reduce fatigability and attentional burden. In this way, many different paths that are specifically tailored on patients’ features could be successfully implemented.

The realization of a P300 BCI-based system, allowing both the cognitive assessment and the development of an AAC tool, could allow to perform a system which fits for user needs; besides, it could also provide useful information for clinicians and caregivers, in order to manage everyday care and future intervention programs.

## References

[b1] Abe K, Fujimura H, Toyooka K, Sakoda S, Yorifuji S, Yanagihara T (1997). Cognitive function in amyotrophic lateral sclerosis. J. Neurol Sci.

[b2] Abrahams S, Goldstein LH, Kew JJ, Brooks DJ, Lloyd CM, Frith CD, Leigh PN (1996). Frontal lobe dysfunction in amyotrophic lateral sclerosis. A PET study. Brain.

[b3] Abrahams S, Goldstein LH, Al-Chalabi A, Pickering A, Morris RG, Passingham RE, Brooks DJ, Leigh PN (1997). Relation between cognitive dysfunction and pseudobulbar palsy in amyotrophic lateral sclerosis. J. Neurol. Neurosurg. Psychiatry.

[b4] Abrahams S, Leigh PN, Harvey A, Vythelingum GN, Grisé D, Goldstein LH (2000). Verbal fluency and executive dysfunction in amyotrophic lateral sclerosis (ALS). Neuropsychologia.

[b5] Abrahams S, Goldstein LH, Suckling J, Ng V, Simmons A, Chitnis X, Atkins L, Williams SC, Leigh PN (2005a). Frontotemporal white matter changes in amyotrophic lateral sclerosis. J. Neurol.

[b6] Abrahams S, Leigh PN, Goldstein LH (2005b). Cognitive change in ALS: a prospective study. Neurology.

[b7] Anastasia A, Urbina S (1997). Psychological testing.

[b8] Bai O, Lin P, Huang D, Fei DY, Floeter MK (2010). Towards a user-friendly brain-computer interface: initial tests in ALS and PLS patients. Clin. Neurophysiol.

[b9] Bak TH, Hodges JR (2004). The effects of motor neurone disease on language: further evidence. Brain Lang.

[b10] Beukelman DR, Fager S, Ball L, Dietz A (2007). AAC for adults with acquired neurological conditions: a review. Augment. Altern. Commun.

[b11] Birbaumer N, Elbert T, Rockstroh B, Lutzenberger W, McCallum WC, Zappoli R, Denoth F (1986). Biofeedback of slow cortical potentials in attentional disorders. Cerebral psychophysiology: studies in event-related potentials.

[b12] Birbaumer N, Ghanayim N, Hinterberger T, Iversen I, Kotchoubey B, Kübler A, Perelmouter J, Taub E, Flor H (1999). A spelling device for the paralysed. Nature.

[b13] Birbaumer N, Hinterberger T, Kübler A, Neumann N (2003). The thought-translation device (TTD): neurobehavioral mechanisms and clinical outcome. IEEE Trans. Neural Syst. Rehabil. Eng.

[b151] Brouwer AM, van Erp JB (2010). A tactile P300 brain-computer interface. Front Neurosci.

[b15] Brownell B, Oppenheimer DR, Hughes JT (1970). The central nervous system in motor neurone disease. J. Neurol. Neurosurg. Psychiatry.

[b16] Brunner P, Joshi S, Briskin S, Wolpaw JR, Bischof H, Schalk G (2010). Does the “P300” speller depend on eye gaze?. J. Neural Eng.

[b17] Cipresso P, Meriggi P, Carelli L, Solca F, Meazzi D, Poletti B, Lule D, Ludolph AC, Riva G, Silani V (2011). The combined use of brain computer interface and eye-tracking technology for cognitive assessment in amyotrophic lateral sclerosis.

[b18] Cipresso P, Meriggi P, Carelli L, Solca F, Poletti B, Lulé D, Ludolph AC, Silani V, Riva G (2012). Brain computer interface and eye-tracking for neuropsychological assessment of executive functions: a pilot study. 10.5220/0003893100790088.

[b19] Citi L, Poli R, Cinel C (2010). Documenting, modelling and exploiting P300 amplitude changes due to variable target delays in Donchin's speller. J. Neural. Eng.

[b20] David AS, Gillham RA (1986). Neuropsychological study of motor neuron disease. Psychosomatics.

[b21] Donchin E (1981). Presidential address, 1980. Surprise!…Surprise?. Psychophysiology.

[b22] Donchin E, Spencer KM, Wijesinghe R (2000). The mental prosthesis: assessing the speed of a P300-based brain-computer interface. IEEE Trans. Rehabil. Eng.

[b23] Doran M, Xuereb JH, Hodges JR (1995). Rapidly progressive aphasia with bulbar motor neurone disease: a clinical and neuropsychological study. Behav. Neurol.

[b24] Duncan-Johnson CC, Donchin E (1982). The P300 component of the event-related brain potential as an index of information processing. Biol. Psychol.

[b25] Eisen A, Krieger C (1993). Pathogenic mechanisms in sporadic amyotrophic lateral sclerosis. Can. J. Neurol Sci.

[b26] Evdokimidis I, Constantinidis TS, Gourtzelidis P, Smyrnis N, Zalonis I, Zis PV, Andreadou E, Papageorgiou C (2002). Frontal lobe dysfunction in amyotrophic lateral sclerosis. J. Neurol Sci.

[b27] Fabiani M, Gratton G, Karis D, Donchin E, Ackles PK, Jennings JR, Coles MGH (1987). Definition, identification and reliability of the P300 component of the event-related brain potential. Advances in psychophysiology.

[b28] Farwell LA, Donchin E (1988). Talking off the top of your head: toward a mental prosthesis utilizing event-related brain potentials. Electroencephalogr. Clin. Neurophysiol.

[b29] Freeman WJ, Holmes MD, Burke BC, Vanhatalo S (2003). Spatial spectra of scalp EEG and EMG from awake humans. Clin. Neurophysiol.

[b30] Furdea A, Halder S, Krusienski DJ, Bross D, Nijboer F, Birbaumer N, Kübler A (2009). An auditory oddball (P300) spelling system for brain-computer interfaces. Psychophysiology.

[b31] Gallagher JP (1989). Pathologic laughter and crying in ALS: a search for their origin. Acta Neurol. Scand.

[b32] Gallassi R, Montagna P, Morreale A, Lorusso S, Tinuper P, Daidone R, Lugaresi E (1989). Neuropsychological, electroencephalogram and brain computed tomography findings in motor neuron disease. Eur. Neurol.

[b33] Gil R, Neau JP, Dary-Auriol M, Agbo C, Tantot AM, Ingrand P (1995). Event-related auditory evoked potentials and amyotrophic lateral sclerosis. Arch. Neurol.

[b171] Girardi A, Macpherson SE, Abrahams S (2011). Deficits in emotional and social cognition in amyotrophic lateral sclerosis. Neuropsychology.

[b35] Gonsalvez CL, Polich J (2002). P300 amplitude is determined by target-to-target interval. Psychophysiology.

[b36] Grosskreutz J, Kaufmann J, Frädrich J, Dengler R, Heinze HJ, Peschel T (2006). Widespread sensorimotor and frontal cortical atrophy in Amyotrophic Lateral Sclerosis. BMC Neurol.

[b37] Grossman AB, Woolley-Levine S, Bradley WG, Miller RG (2007). Detecting neurobehavioral changes in amyotrophic lateral sclerosis. Amyotroph. Lateral Scler.

[b38] Guger C, Edlinger G, Harkam W, Niedermayer I, Pfurtscheller G (2003). How many people are able to operate an EEG-based brain-computer interface (BCI)?. IEEE Trans. Neural Syst. Rehabil. Eng.

[b39] Guger C, Daban S, Sellers E, Holzner C, Krausz G, Carabalona R, Gramatica F, Edlinger G (2009). How many people are able to control a P300-based brain-computer interface (BCI)?. Neurosci. Lett.

[b40] Hagen GF, Gatherwright JR, Lopez BA, Polich J (2006). P3a from visual stimuli: task difficulty effects. Int. J. Psychophysiol.

[b41] Han J, Ma L (2010). Study of the features of proton MR spectroscopy ((1)H-MRS) on amyotrophic lateral sclerosis. J. Magn. Reson. Imaging.

[b42] Hanagasi HA, Gurvit IH, Ermutlu N, Kaptanoglu G, Karamursel S, Idrisoglu HA, Emre M, Demiralp T (2002). Cognitive impairment in amyotrophic lateral sclerosis: evidence from neuropsychological investigation and event-related potentials. Brain Res. Cogn. Brain Res.

[b43] Hill NJ, Lal TN, Schröder M, Hinterberger T, Wilhelm B, Nijboer F, Mochty U, Widman G, Elger C, Schölkopf B (2006). Classifying EEG and ECoG signals without subject training for fast BCI implementation: comparison of nonparalyzed and completely paralyzed subjects. IEEE Trans. Neural Syst. Rehabil. Eng.

[b44] Hinterberger T, Veit R, Wilhelm B, Weiskopf N, Vatine JJ, Birbaumer N (2005). Neuronal mechanisms underlying control of a brain-computer interface. Eur. J. Neurosci.

[b45] Hoffmann U, Vesin JM, Ebrahimi T, Diserens K (2008). An efficient P300-based brain-computer interface for disabled subjects. J. Neurosci. Methods.

[b46] Irwin D, Lippa CF, Swearer JM (2007). Cognition and amyotrophic lateral sclerosis (ALS). Am. J. Alzheimers Dis. Other Demen.

[b47] Iversen IH, Ghanayim N, Kübler A, Neumann N, Birbaumer N, Kaiser J (2008a). A brain-computer interface tool to assess cognitive functions in completely paralyzed patients with amyotrophic lateral sclerosis. Clin. Neurophysiol.

[b48] Iversen I, Ghanayim N, Kübler A, Neumann N, Birbaumer N, Kaiser J (2008b). Conditional associative learning examined in a paralyzed patient with amyotrophic lateral sclerosis using brain-computer interface technology. Behav. Brain Funct.

[b49] Iwasaki Y, Kinoshita M, Ikeda K, Takamiya K, Shiojima T (1990). Cognitive impairment in amyotrophic lateral sclerosis and its relation to motor disabilities. Acta Neurol. Scand.

[b50] Jokelainen M (1977). Amyotrophic lateral sclerosis in Finland. I: an epidemiologic study. Acta Neurol. Scand.

[b51] Kew JJ, Goldstein LH, Leigh PN, Abrahams S, Cosgrave N, Passingham RE, Frackowiak RS, Brooks DJ (1993). The relationship between abnormalities of cognitive function and cerebral activation in amyotrophic lateral sclerosis. A neuropsychological and positron emission tomography study. Brain.

[b52] Kleih SC, Nijboer F, Halder S, Kübler A (2010). Motivation modulates the P300 amplitude during brain –computer interface use. Clin. Neurophysiol.

[b53] Klobassa DS, Vaughan TM, Brunner P, Schwartz NE, Wolpaw JR, Neuper C, Sellers EW (2009). Toward a high-throughput auditory P300-based brain-computer interface. Clin. Neurophysiol.

[b54] Kotchoubey B (1997). A new method for self-regulation of slow cortical potentials in a timed paradigm. Appl. Psychophysiol. Biofeedback.

[b55] Krusienski DJ, Sellers EW, Cabestaing F, Bayoudh S, McFarland DJ, Vaughan TM, Wolpaw JR (2006). A comparison of classification techniques for the P300 Speller. J. Neural. Eng.

[b56] Kübler A, Birbaumer N (2008). Brain-computer interfaces and communication in paralysis: extinction of goal directed thinking in completely paralysed patients?. Clin. Neurophysiol.

[b57] Kubler A, Muller KR, Nijholt A, Tan DS, Pfurtscheller G, Brunner C, del R. Millán J, Allison B, Graimann B, Popescu F, Blankertz B, Möller K-R (2007). Toward brain-computer interfacing. Brain computer interfacing for intelligent systems.

[b58] Kübler A, Kotchoubey B, Hinterberger T, Ghanayim N, Perelmouter J, Schauer M, Fritsch C, Taub E, Birbaumer N (1999). The thought translation device: a neurophysiological approach to communication in total motor paralysis. Exp. Brain Res.

[b59] Kübler A, Neumann N, Kaiser J, Kotchoubey B, Hinterberger T, Birbaumer NP (2001a). Brain-computer communication: self-regulation of slow cortical potentials for verbal communication. Arch. Phys. Med. Rehabil.

[b60] Kübler A, Kotchoubey B, Kaiser J, Wolpaw JR, Birbaumer N (2001b). Brain-computer communication: unlocking the locked in. Psychol. Bull.

[b61] Kübler A, Nijboer F, Mellinger J, Vaughan TM, Pawelzik H, Schalk G, McFarland DJ, Birbaumer N, Wolpaw JR (2005). Patients with ALS can use sensorimotor rhythms to operate a brain-computer interface. Neurology.

[b62] Kwong LK, Neumann M, Sampathu DM, Lee VM, Trojanowski JQ (2007). TDP-43 proteinopathy: the neuropathology underlying major forms of sporadic and familial frontotemporal lobar degeneration and motor neuron disease. Acta Neuropathol.

[b63] Leuthardt EC, Schalk G, Roland J, Rouse A, Moran DW (2009). Evolution of brain-computer interfaces: going beyond classic motor physiology. Neurosurg. Focus.

[b64] Lomen-Hoerth C, Murphy J, Langmore S, Kramer JH, Olney RK, Miller B (2003). Are amyotrophic lateral sclerosis patients cognitively normal?. Neurology.

[b65] Ludolph AC, Langen KJ, Regard M, Herzog H, Kemper B, Kuwert T, Böttger IG, Feinendegen L (1992). Frontal lobe function in amyotrophic lateral sclerosis: a neuropsychologic and positron emission tomography study. Acta Neurol. Scand.

[b66] Lulé D, Kurt A, Jürgens R, Kassubek J, Diekmann V, Kraft E, Neumann N, Ludolph AC, Birbaumer N, Anders S (2005). Emotional responding in amyotrophic lateral sclerosis. J. Neurol.

[b204] Lulé D, Diekmann V, Anders S, Kassubek J, Kübler A, Ludolph AC, Birbaumer N (2007). Brain responses to emotional stimuli in patients with amyotrophic lateral sclerosis (ALS). J. Neurol.

[b68] Lule D, Diekmann V, Muller H, Kassubek J, Ludolph AC, Birbaumer N (2010). Neuroimaging of multimodal sensory stimulation in amyotrophic lateral sclerosis. J. Neurol. Neurosurg. Psychiatry.

[b69] Lutzenberger W, Birbaumer N, Elbert T, Rockstroh B, Bippus W, Breidt R (1980). Self-regulation of slow cortical potentials in normal subjects and patients with frontal lobe lesions. Prog. Brain Res.

[b70] Lutzenberger W, Roberts LE, Birbaumer N (1993). Memory performance and area-specific self-regulation of slow cortical potentials: dual-task interference. Int. J. Psychophysiol.

[b71] Mantovan MC, Baggio L, Dalla Barba G, Smith P, Pegoraro E, Soraru' G, Bonometto P, Angelini C (2003). Memory deficits and retrieval processes in ALS. Eur. J. Neurol.

[b72] Martens SMM, Hill NJ, Farquhar J, Schölkopf B (2009). Overlap and refractory effects in a brain-computer interface speller based on the visual P300 event-related potential. J. Neural Eng.

[b73] Massman PJ (1996). Prevalence and correlates of neuropsychological deficits in amyotrophic lateral sclerosis. J. Neurol. Neurosurg. Psychiatry.

[b74] McDonald JJ, Teder-Salejarvi WA, Hillyard SA (2000). Involuntary orienting to sound improves visual perception. Nature.

[b75] McFarland DJ, Miner LA, Vaughan TM, Wolpaw JR (2000). Mu and beta rhythm topographies during motor imagery and actual movements. Brain Topogr.

[b76] Mohammadi B, Kollewe K, Samii A, Dengler R, Münte TF (2011). Functional neuroimaging at different disease stages reveals distinct phases of neuroplastic changes in amyotrophic lateral sclerosis. Hum. Brain Mapp.

[b77] Murphy JM, Henry RG, Langmore S, Kramer JH, Miller BL, Lomen-Hoerth C (2007). Continuum of frontal lobe impairment in amyotrophic lateral sclerosis. Arch. Neurol.

[b78] Neumann M, Sampathu DM, Kwong LK, Truax AC, Micsenyi MC, Chou TT, Bruce J, Schuck T, Grossman M, Clark CM, McCluskey LF, Miller BL, Masliah E, Mackenzie IR, Feldman H, Feiden W, Kretzschmar HA, Trojanowski JQ, Lee VM (2006). Ubiquitinated TDP-43 in frontotemporal lobar degeneration and amyotrophic lateral sclerosis. Science.

[b79] Newsom-Davis IC, Abrahams S, Goldstein LH, Leigh PN (1999). The emotional lability questionnaire: a new measure of emotional lability in amyotrophic lateral sclerosis. J. Neurol. Sci.

[b80] Niedermeyer E, Niedermeyer E, Lopes da Silva F (2004). The normal EEG of the waking adult. Electroencephalography: basic principles, clinical applications and related fields.

[b81] Nijboer F, Sellers EW, Mellinger J, Jordan MA, Matuz T, Furdea A, Halder S, Mochty U, Krusienski DJ, Vaughan TM, Wolpaw JR, Birbaumer N, Kübler A (2008). A P300-based brain-computer interface for people with amyotrophic lateral sclerosis. Clin. Neurophysiol.

[b82] Nijboer F, Birbaumer N, Kubler A (2010). The influence of psychological state and motivation on brain-computer interface performance in patients with amyotrophic lateral sclerosis - a longitudinal study. Front Neurosci.

[b83] Norani NAM, Mansor W, Khuan LY (2010). A review of signal processing in brain computer interface system.

[b84] Ogawa T, Tanaka H, Hirata K (2009). Cognitive deficits in amyotrophic lateral sclerosis evaluated by event-related potentials. Clin. Neurophysiol.

[b85] Papps B, Abrahams S, Wicks P, Leigh PN, Goldstein LH (2005). Changes in memory for emotional material in amyotrophic lateral sclerosis (ALS). Neuropsychologia.

[b86] Paulus KS, Magnano I, Piras MR, Solinas MA, Solinas G, Sau GF, Aiello I (2002). Visual and auditory event-related potentials in sporadic amyotrophic lateral sclerosis. Clin. Neurophysiol.

[b87] Perego P, Turconi AC, Andreoni G, Maggi L, Beretta E, Parini S, Gagliardi C (2011). Cognitive ability assessment by brain-computer interface validation of a new assessment method for cognitive abilities. J. Neurosci. Methods.

[b88] Perelmouter J, Birbaumer N (2000). A binary spelling interface with random errors. IEEE Trans. Rehabil. Eng.

[b89] Perelmouter J, Kotchoubey B, Kübler A, Birbaumer N (1999). Language support program for thought-translation devices. Automedica.

[b90] Pfurtscheller G, Niedermeyer E, d. Silva FL (1999). EEG event-related desynchronization (ERD) and event-related synchronization (ERS). Electroencephalography: basic principles, clinical applications and related fields.

[b91] Pfurtscheller G, Neuper C (1997). Motor imagery activates primary sensorimotor area in humans. Neurosci. Lett.

[b92] Pfurtscheller G (2000). Current trends in Graz Brain-Computer Interface (BCI) research. IEEE Trans. Rehabil. Eng.

[b93] Phukan J, Pender NP, Hardiman O (2007). Cognitive impairment in amyotrophic lateral sclerosis. Lancet Neurol.

[b94] Phukan J, Elamin M, Bede P, Jordan N, Gallagher L, Byrne S, Lynch C, Pender N, Hardiman O (2011). The syndrome of cognitive impairment in amyotrophic lateral sclerosis: a population-based study. J. Neurol. Neurosurg. Psychiatry.

[b95] Pinkhardt EH, Jürgens R, Becker W, Mölle M, Born J, Ludolph AC, Schreiber H (2008). Signs of impaired selective attention in patients with amyotrophic lateral sclerosis. J. Neurol.

[b96] Polich J (2004). Clinical application of the P300 event-related brain potential. Phys. Med. Rehabil. Clin. N. Am.

[b97] Polich J (2007). Updating P300: an integrative theory of P3a and P3b. Clin. Neurophysiol.

[b98] Polich J, McIsaac HK (1994). Comparison of auditory P300 habituation from active and passive conditions. Int. J. Psychophysiol.

[b99] Rakowicz WP, Hodges JR (1998). Dementia and aphasia in motor neuron disease: an underrecognised association?. J. Neurol. Neurosurg. Psychiatry.

[b100] Ringholz GM, Appel SH, Bradshaw M, Cooke NA, Mosnik DM, Schulz PE (2005). Prevalence and patterns of cognitive impairment in sporadic ALS. Neurology.

[b101] Rippon GA, Scarmeas N, Gordon PH, Murphy PL, Albert SM, Mitsumoto H, Marder K, Rowland LP, Stern Y (2006). An observational study of cognitive impairment in amyotrophic lateral sclerosis. Arch. Neurol.

[b102] Robinson KM, Lacey SC, Grugan P, Glosser G, Grossman M, McCluskey LF (2006). Cognitive functioning in sporadic amyotrophic lateral sclerosis: a six month longitudinal study. J. Neurol. Neurosurg. Psychiatry.

[b103] Sach M, Winkler G, Glauche V (2004). Diffusion tensor MRI of early upper motor neuron involvement in amyotrophic lateral sclerosis. Brain.

[b104] Sage CA, Peeters RR, Görner A, Robberecht W, Sunaert S (2007). Quantitative diffusion tensor imaging in amyotrophic lateral sclerosis. Neuroimage.

[b105] Salvaris M, Sepulveda F (2009). Perceptual errors in the Farwell and Donchin matrix speller.

[b106] Schneider F, Rockstroh B, Heimann H, Lutzenberger W, Mattes R, Elbert T, Birbaumer N, Bartels M (1992). Self-regulation of slow cortical potentials in psychiatric patients: schizophrenia. Biofeedback Self-Regul.

[b107] Schreiber H, Gaigalat T, Wiedemuth-Catrinescu U, Graf M, Uttner I, Muche R, Ludolph AC (2005). Cognitive function in bulbar- and spinal-onset amyotrophic lateral sclerosis. A longitudinal study in 52 patients. J. Neurol.

[b108] Sellers EW, Donchin E (2006). A P300-based brain-computer interface: initial tests by ALS patients. Clin. Neurophysiol.

[b109] Sellers EW, Kubler A, Donchin E (2006). Brain-computer interface research at the University of South Florida Cognitive Psychophysiology Laboratory: the P300 Speller. IEEE Trans. Neural Syst. Rehabil. Eng.

[b110] Senda J, Ito M, Watanabe H (2009). Correlation between pyramidal tract degeneration and widespread white matter involvement in amyotrophic lateral sclerosis: a study with tractography and diffusion-tensor imaging. Amyotroph. Lateral Scler.

[b111] Serby H, Yom-Tov E, Inbar GF (2005). An improved P300-based brain-computer interface. IEEE Trans. Neural Syst. Rehabil. Eng.

[b112] Silvoni S, Volpato C, Cavinato M, Marchetti M, Priftis K, Merico A, Tonin P, Koutsikos K, Beverina F, Piccione F (2009). P300-based brain-computer interface communication: evaluation and follow-up in amyotrophic lateral sclerosis. Front Neurosci.

[b113] Squires NK, Donchin E, Squires KC, Grossberg S (1977). Bisensory stimulation: inferring decision-related processes from P300 component. J. Exp. Psychol. Hum. Percept Perform.

[b114] Srinivasan R, Nunez PL, Silberstein RB (1998). Spatial filtering and neocortical dynamics: estimates of EEG coherence. IEEE Trans. Biomed. Eng.

[b115] Stanton BR, Williams VC, Leigh PN, Williams SC, Blain CR, Jarosz JM, Simmons A (2007). Altered cortical activation during a motor task in ALS. Evidence for involvement of central pathways. J. Neurol.

[b116] Strong M, Rosenfeld J (2003). Amyotrophic lateral sclerosis: a review of current concepts. Amyotroph. Lateral Scler. Other Motor Neuron Disord.

[b117] Strong MJ, Grace GM, Orange JB, Leeper HA (1996). Cognition, language, and speech in amyotrophic lateral sclerosis: a review. J. Clin. Exp. Neuropsychol.

[b118] Strong MJ, Grace GM, Orange JB, Leeper HA, Menon RS, Aere C (1999). A prospective study of cognitive impairment in ALS. Neurology.

[b119] Strong MJ, Yang W, Strong WL, Leystra-Lantz C, Jaffe H, Pant HC (2006). Tau protein hyperphosphorylation in sporadic ALS with cognitive impairment. Neurology.

[b120] Sutter EE (1992). The brain response interface: communication through visually-induced electrical brain responses. J. Microcomput. Appl.

[b121] Sutton S, Braren M, Zubin J, John ER (1965). Evoked-potential correlates of stimulus uncertainty. Science.

[b122] Talbot PR, Goulding PJ, Lloyd JJ, Snowden JS, Neary D, Testa HJ (1995). Inter-relation between “classic” motor neuron disease and frontotemporal dementia: neuropsychological and single photon emission computed tomography study. J. Neurol Neurosurg. Psychiatry.

[b123] Tan D, Nijholt A (2010). Brain-computer interface and human-computer interaction. Brain-computer interfaces: applying our minds to human-computer interaction, 2010.

[b124] Tanaka M, Kondo S, Hirai S, Sun X, Yamagishi T, Okamoto K (1993). Cerebral blood flow and oxygen metabolism in progressive dementia associated with amyotrophic lateral sclerosis. J. Neurol. Sci.

[b125] Teder-Sälejärvi WA, McDonald JJ, Di Russo F, Hillyard SA (2002). An analysis of audio-visual crossmodal integration by means of event-related potential (ERP) recordings. Brain Res. Cogn. Brain Res.

[b126] Teplan N (2002). Fundamentals of EEG measurement. Meas. Sci. Rev.

[b127] Treder MS, Blankertz B (2010). (C)overt attention and visual speller design in an ERP-based brain-computer interface. Behav. Brain Funct.

[b128] Tsermentseli S, Leigh PN, Goldstein LH (2012). The anatomy of cognitive impairment in amyotrophic lateral sclerosis: more than frontal lobe dysfunction. Cortex.

[b129] Turner M, Hammers A, Allsop J (2007). Volumetric cortical loss in sporadic and familial amyotrophic lateral sclerosis. Amyotroph. Lateral Scler.

[b130] Vaughan TM, McFarland DJ, Schalk G, Sarnacki WA, Krusienski DJ, Sellers EW, Wolpaw JR (2006). The Wadsworth BCI Research and Development Program: at home with BCI. IEEE Trans. Neural Syst. Rehabil. Eng.

[b131] Verleger R (2008). P3b: towards some decision about memory. Clin. Neurophysiol.

[b132] Vidal JJ (1977). Real-time detection of brain events in EEG. Biol. Signal Process. Analysis.

[b133] Vieregge P, Wauschkuhn B, Heberlein I, Hagenah J, Verleger R (1999). Selective attention is impaired in amyotrophic lateral sclerosis–a study of event-related EEG potentials. Brain Res. Cogn. Brain Res.

[b134] Wolpaw JR, McFarland DJ (2004). Control of a two-dimensional movement signal by a noninvasive brain-computer interface in humans. Proc. Natl. Acad. Sci. U.S.A.

[b135] Wolpaw JR, McFarland DJ, Neat GW, Forneris CA (1991). An EEG-based brain-computer interface for cursor control. Electroencephalogr. Clin. Neurophysiol.

[b136] Wolpaw JR, Birbaumer N, Heetderks WJ, McFarland DJ, Peckham PH, Schalk G, Donchin E, Quatrano LA, Robinson CJ, Vaughan TM (2000). Brain-computer interface technology: a review of the first international meeting. IEEE Trans. Rehabil. Eng.

[b137] Wolpaw JR, Birbaumer N, McFarland DJ, Pfurtscheller G, Vaughan TM (2002). Brain-computer interfaces for communication and control. Clin. Neurophysiol.

